# Comparative Analysis of the Polyphenols, Caffeine, and Antioxidant Activities of Green Tea, White Tea, and Flowers from Azorean *Camellia sinensis* Varieties Affected by Different Harvested and Processing Conditions

**DOI:** 10.3390/antiox10020183

**Published:** 2021-01-27

**Authors:** Lisete Paiva, Clara Rego, Elisabete Lima, Massimo Marcone, José Baptista

**Affiliations:** 1 Chemistry and Engineering (DPCE) and Biotechology Centre of Azores (CBA), Department of Physics, University of Azores, 9500-321 Ponta Delgada, São Miguel, Azores, Portugal; lisete.s.paiva@uac.pt (L.P.); jose.ab.baptista@uac.pt (J.B.); 2 Institute of Agricultural and Environmental Research and Technology (IITAA), University of Azores, 9700-042 Angra do Heroísmo, Terceira, Azores, Portugal; 3 São Miguel Agrarian Development Service (SDASM), 9500-340 Ponta Delgada, São Miguel, Azores, Portugal; c.estrela5@gmail.com; 4 Department of Food Science, University of Guelph, Guelph, ON N1G 2W1, Canada; mmarcone@uoguelph.ca

**Keywords:** green/white tea, flowers waste valorization, antioxidant activity, phenolic/flavonoid content, catechin and caffeine profiles, plucking and processing variability, RP-HPLC/PDAD analysis

## Abstract

This study evaluates the polyphenol profiles as well as caffeine (dry weight basis), and antioxidant activities of green tea (GTs), white tea (WTs), and flowers (Fl) samples from Azorean *Camellia sinensis* varieties affected by different harvested and processing conditions. Epicatechins derivatives, determined by RP-HPLC/PDAD, presented higher values in GTs with respect to WTs, decreasing as follows: epigallocatechin-3-gallate > epicatechin-3-gallate ≫ epicatechin ≫ epigallocatechin, and higher values in summer and early autumn than in spring. This was also accompanied by an in consistent withering time pattern. Esterified catechins were higher in all samples (100.8–312.3 mg/g) with respect to non-esterified catechins (15.1–37.7 mg/g). Caffeine (6.2–27.7 mg/g) decreased as follows: WTs > GTs ≫ Fl, and inconsistent seasonal and withering patterns were observed among the WTs. Total phenolics (125.9–295.4 mg gallic acid equivalents/g dried extract) and total flavonoids (35.2–69.7 mg rutin equivalents/g dried extract), determined by Folin–Ciocalteu and colorimetric methodologies, were higher in GTs than in WTs and Fl. Concerning the antioxidant patterns, the free radical scavenging activity (FRSA) and ferric reducing antioxidant power (FRAP) presented EC_50_ values ranges from 3.6 to 17.3 µg/mL and 4.8 to 16.5 µg/mL, respectively, and ferrous ion-chelating (FIC) activity ranged from 47.1 to 82.8%, highlighting that FRSA was better than butylated hydroxytoluene (BHT). Tea leaves exhibited, in general, higher activities with respect to tea Fl, and the WT sample plucked in summer and withered for 23 h showed the highest FRAP and FIC activity. In conclusion, this study shows the characteristic variation of GTs, WTs, and Fl of two tea varieties and may support crop quality improvement and promote the valorization of tea Fl.

## 1. Introduction

The drink, green tea (GT), made from *Camellia sinensis* L. tea leaves is one of the oldest and most widely consumed non-alcoholic beverages worldwide following water, owing to its pleasant sensory characteristics, scientifically proven beneficial effects on human health, unique sociocultural characteristics [1], stimulant properties, and relatively low retail price. The tea plant, native to Southeast China, gradually expanded into many tropical and subtropical countries [2] and, since the last decade of the 19th century, it is also commercially produced in one unique place in Europe, the volcanic São Miguel Island of the Azores Archipelago (Portugal). The organic tea is cultivated in the Azores as a sustainable and eco-friendly approach in order to enrich quality of life and improve human health. Tea polyphenols (TPs), particularly the catechins group, are considered key contributors to the protective effects that GT possess against several diseases (e.g., cancer, cardiovascular disease, liver disease, type 2 diabetes, and neurodegenerative diseases) as well as the ageing process, due to their diverse pharmacological activities, in particular, their strong antioxidant properties (for recent reviews, see [3,4,5,6]). In addition to their antioxidant activity, tea catechins have anti-inflammatory properties and have been shown to have an effect on the increased uptake of glucocorticoids in lungs diseases and, thus, tea catechins supplementation may be also beneficial as therapeutic strategies against COVID-19 [7,8]. *C. sinensis* young shoots (also referred to as fresh green leaves) are also widely known for their valuable functional molecules, such as L-theanine that is used as one of the biosynthetic precursors of catechins [9]. According to the literature, it is estimated that TPs (including flavanols, flavonols, flavones, proanthocyanins, and phenolic acids) account for approximately 20–35% of the tea flush dry weight (DW), among which 60–80% are catechins (flavan-3-ols). Among them, it is known that epicatechin derivatives (ECDs) are the most important group and can represent approximately 90% of the total catechin content (TCC). Another important functional metabolite in tea is caffeine (CAF), accounting for approximately 2–5% of the tea flush DW [10,11,12]. However, it is well established that the composition of commercial teas can be significantly affected by many factors, such as the plant variety, leaf age, origin of tea production, season, climate, agronomic management, tea processing, and storage (for recent reviews see [6,13]). It is estimated that approximately 78% of the world’s tea production is accounted by black tea, 20% by green tea, and only 2% by other tea types. However, white tea (WT) is becoming more common in Europe due to its delicate and sweet flavor [14,15] and is commercially available in several grades, such as “Pai-Mu-Tan” (White Peony) [14,16]. GT and WT, which are considered to be unfermented, are amongst the least processed teas and this enables them to retain a higher concentration of TPs in their intact form. However, the lengthy drying process under the sun, used for WT processing, leads to slight oxidation of some catechins. In addition, a slight degree of fermentation may also occur given that, in general, WT processing lacks the step of inactivating the polyphenol oxidase enzyme [1,6,14,17,18]. As is well known, each tea type has a unique and crucial manufacture withering process [19] that involves both physical and chemical changes in the plucked tea leaves, which have a resultant impact on tea quality. The appropriate conditions for withering, such as time, temperature, and relative humidity, can vary depending on climate, the producing region, and type of manufacturing process used [20].

Given that TPs and CAF are among the principal metabolites responsible for the various bioactivities and distinctive sensory properties of tea [1], the aim of the present study was to compare, for the first time, the biochemical profiles (individual catechins, CAF, total phenolics, and total flavonoids contents) and antioxidant activities (free radical scavenging activity, ferric reducing antioxidant power, and ferrous ion-chelating activity) of five “Pai-Mu-Tan” WTs (WT1–WT5) at various plucking and processing conditions, with flowers obtained from Azorean *C. sinensis*, var. *assamica* (CSA) cultivated in Sete Cidades location, and with the same number of commercial GTs of Azorean *C. sinensis*, var. *sinensis* (CSS) from Gorreana Tea Plantation, processed under the same conditions. The results of this study will support the selection of those tea leaves which result in improved crop quality as well as maximize the beneficial effects on human health. Moreover, they can also promote the valorization of Azorean *C. sinensis* flowers as a potential biochemical with nutraceutical benefits. The findings of the current study can also contribute to those databases which aim to comprehensively characterize the WT type [16].

## 2. Material and Methods

### 2.1. Chemicals and Reagents

Catechins, namely, (+)-catechin (C, 98%–C1251), (–)-epicatechin (EC, 98%–E4018), (–)-epigallocatechin (EGC, 98%–E3768), (–)-epigallocatechin-3-gallate (EGCG, 95%–E4143), (–)-epicatechin-3-gallate (ECG, 98%–E3893), (+)-gallocatechin (GC, 98%–G6657) and (–)-gallocatechin-3-gallate (GCG, 98%–G6782), caffeine (CAF, 99%–C0750), gallic acid (98%–G7384), rutin, 2,2-diphenyl-1-picrylhydrazyl (DPPH), butylated hydroxytoluene (BHT), ethylenediaminetetraacetic disodium salt (EDTA), Folin–Ciocalteu reagent (FCR), potassium ferricyanide, iron (II) chloride (FeCl_2_), iron (III) chloride (FeCl_3_), aluminum chloride (AlCl_3_), ferrozine, and trichloroacetic acid (TCA) were all obtained from Sigma–Aldrich (St. Louis, MO, USA). Sodium carbonate (Na_2_CO_3_), potassium acetate (KCH_3_CO_2_), sodium phosphate, and orthophosphoric acid were obtained from E. Merck (Darmstadt, Hessen, Germany). Acetonitrile, methanol, chloroform, and ethyl acetate, HPLC-grade, were obtained from Riedel-de Häen (Aktiengesellschaft, Seelze, Germany). Ultrapure glass distilled water that was deionized with the Millipore Milli-Q purification system (Millipore, Bedford, MA, USA) was used throughout all the experiments.

### 2.2. Sample Origin, Withering, and Drying Methodologies

A total of ten tea samples (5 WTs and 5 GTs) plus one flowers sample from two different varieties of Azorean *C. sinensis* were analyzed and their identities are listed in Table 1. The GTs of CSS were provided by Gorreana Tea Plantation (São Miguel Island, Azores, Portugal), while the WTs (type “Pai-Mu-Tan”) and flower sample of CSA from the Sete Cidades location were provided by Experimental Station (São Miguel Agrarian Development Service) under supervision of the Azorean Regional Government (São Miguel Island, Azores, Portugal). The fresh leaves of GTs, under the same processing conditions, were indoor-withered for several hours at 25–30 °C to achieve a relative humidity of 70%, and then heated with boiling water vapor to inactivate the polyphenol oxidase enzyme that causes oxidation. These were then finally dried in a heating chamber at 70 °C for 30 min. The fresh leaves of the WTs (WT1 to WT5) were allowed to wither for several hours (W1 to WT4 at 18 °C in an air acclimatized room and WT5 at 22 °C under a fan for air circulation) and then dried in a solar chamber for several hours. The freshly plucked flowers sample from CSA was also dried in a solar chamber until a constant weight was produced. The protocol for the total TPs and CAF extractions was a slightly modified version of the methodology described by Baptista et al. [21].

### 2.3. Sample Preparation for Antioxidant Assays and Extraction Methodology for Crude Catechins and CAF Content

Knowing the impact of sample preparation conditions (e.g., solvent type, temperature of extraction, contact time, particle size, and solute:solvent ratio) on the preservation of antioxidant power [21,22] and tea extraction yield, as well as to mimic the tea infusions used by general people, our aqueous TPs (the major soluble components in a cup of tea) extraction procedure was carried out as follows: each dried sample was grounded in a mortar to a particle size of 20–30 µm and 1 g of the powdered material was extracted with water (3 × 20 mL) at 70 °C for 15 min, with stirring, under an atmosphere of N_2_ to prevent oxidation. The solution of the combined extracts was filtered under vacuum through a 0.45 µm (pore size) cellulose acetate membrane to remove particulate matter, freeze-dried after vacuum rotaevaporator concentration, and stored prior to evaluation of their antioxidant activities and further analysis. A water solution of the freeze-dried tea sample at 1 and 2 mg/mL concentrations were used for antioxidant assays and total phenolics and total flavonoids determinations, respectively. The extraction of crude catechins and CAF was performed according to the method published by Baptista et al. [22] with slight modifications. A 100 mg sample (freeze-dried tea powder) was extracted with water using the methodology described above, concentrated in a vacuum rotaevaporator, reconstituted in 25 mL (volumetric flask) of distilled water, and a volume of 10 mL was partitioned with an equal volume of chloroform to remove pigments and other non-polar plant material. Then, the aqueous layer was extracted with ethyl acetate (3 × 10 mL) to obtain the catechins mixture. The solution of the combined ethyl acetate extracts was evaporated in a vacuum rotaevaporator and the light-brown residue (crude catechins) was dissolved in 500 µL of water and then subjected to high-performance liquid chromatography/photodiode array detection (RP-HPLC/PDAD) analysis after being filtered through a 0.45 𝜇m polytetra-fluoroethylene membrane cartridge.

### 2.4. RP-HPLC Analysis of Catechins and CAF

Variability in terms of the catechin profiles and CAF content in the samples was determined by RP-HPLC/PDAD because it provides an efficient and high-throughput analysis methodology for separation and quantification of these tea components, as validated in our previous research on GT [22] and tea extracts [21]. Since the aromatic structural similarity of the tea catechins made the separation difficult, an Ultropac Spherisorb ODS2 column (100 × 4.6 mm i.d.) from LKB (Bromma, Sweden) was used to separate the individual catechins with high resolution, particularly between EC and EGCG, due to its hydrophobicity, carbon number (12%), and small particle size (3 μm). The mobile phase was composed by acetonitrile:ethyl acetate:0.1% orthophophoric acid:water (4.25:1:44.75:50, *v*/*v*/*v*/*v*) as mobile phase A, and acetonitrile:water (1:1, *v*/*v*) as mobile phase B. Baseline separation was achieved with a gradient elution as follows: 100% A for 10 min, followed by a linear gradient between phase A and phase B, at an increasing rate of 2% per min of phase B until 20% B is reached, with this composition being maintained until the end of the run at a flow rate of 0.8 mL/min. The injection volume was 10 µL and the total run time was around 34 min. The column was maintained at temperature (35 °C), attached to an Agilent Technologies (Palo Alto, CA, USA) series 1200 Liquid Chromatograph equipped with a PDAD fixed at 280 nm. The chromatograms were recorded according to the retention time (RT), and the quantitative analysis was achieved by the external standard method using the ChemStation Chromatography Software from Agilent Technologies. The sample concentration was limited to the range of linearity in order to avoid peak tailing and RT shifting, which may occur when the sample amount approaches the column sample load capacity. Peak identity was performed by RT based on comparison with the authentic standards and/or by spiking the sample with the same standards. Individual catechins were also confirmed by superimposing the spectrum of the peak with the corresponding authentic standard spectrum. The multilevel working calibration curves at five different concentrations, plus the linear range in µmol/L for the major catechins derivatives (ECDs), were determined, as well as the limit of detection (LOD) and limit of quantification (LOQ), which are defined as the amount of injected sample that give a signal to noise ratio of 3 and 10, respectively.

The average of the triplicate measurements was used to calculate the catechin and CAF content and were expressed as mg/g of the sample mass on a DW basis. The total ECDs, total esterified catechins, and total non-esterified catechins were obtained by summation as follows: EC + EGC + EGCG + ECG; EGCG + ECG + GCG and C + EC + EGC + GC, respectively.

### 2.5. Determination of the Total Phenolic and Total Flavonoid Content of TPs Extracts

Total phenolic content (TPC) was determined by using Folin–Ciocalteu colorimetric methodology based on the oxidation/reduction reaction as described by Waterhouse [23], with some modifications [24]. An aliquot of 100 µL of each extract sample (2 mg/mL) was mixed with 1500 µL of distilled water and 100 µL of 2N FCR, homogenized in a vortex for 15 s, and placed in dark for 3 min. Then, 300 µL of 10% Na_2_CO_3_ (*w*/*v*) was added to the mixture, homogenized and incubated for 5 min at 50 °C. The absorbance (Abs) of samples was measured at 760 nm. Gallic acid was used to produce a standard calibration curve at various concentrations and the results were expressed in milligrams of gallic acid equivalents per gram of dried extract (mg GAE/g of DE).

To determine total flavonoid content (TFC), the colorimetric method of Chang et al. [25] was used with some modifications [24]. An aliquot of 100 µL of each extract sample (2 mg/mL) was mixed with 100 µL of 10% AlCl_3_, 100 µL of 10% KCH_3_CO_2_, plus 900 µL of distilled water. The mixture was homogenized in a vortex for 15 s, and the Abs was measured at 415 nm after 30 min at room temperature. Rutin was used to produce a standard calibration curve at various concentrations and the results were expressed as mg of rutin equivalents per gram of dried extract (mg RE/g of DE).

### 2.6. Determination of the in Vitro Antioxidant Activity of TPs Extracts

Assessing the antioxidant properties of bioactive natural compounds is highly important due to the high commercial interest of natural antioxidants for pharmaceutical, nutraceutical, cosmetic, and food industries, especially. To take into account the various mechanisms by which plant antioxidants can exert their action, the infusions under study were evaluated using different in vitro antioxidant assays, such as: DPPH-FRSA, FRAP, and FIC activity.

#### 2.6.1. Determination of DPPH-Free Radical Scavenging Activity (FRSA)

The DPPH-FRSA assay, based on both electron transfer and hydrogen atom transfer reactions, was determined according to the method of Molyneux [26] with slight modifications [24]. The FRSA of each freeze-dried extract at various concentrations was tested by measuring their ability to quench DPPH. The DPPH, a stable free radical, was reduced, causing the purple color of the DPPH solution to change to bright yellow in the presence of antioxidants that possess hydrogen-donating or chain-breaking properties, and the intensity of this change can be monitored spectrophotometrically. An aliquot of 250 µL of each extract sample (concentration range 3.33–20 µg/mL) or BHT was added to 500 µL of 100 µM DPPH solution. BHT was used as the reference sample at the same concentration of the extracts and a mixture without sample, or BHT, was used as the control. The Abs was measured at 517 nm after incubation (at room temperature in the dark) for a period of 30 min. The FRSA was calculated as a percentage of DPPH decoloration using the following equation:

FRSA (%) = (1 − Abs_sample_/Abs_control)_ × 100 (1)

The results were expressed as EC_50_ value (µg/mL), which is defined as the sample concentration that can quench 50% of the DPPH free radicals. A lower EC_50_ value was indicative of a higher antioxidant activity.

#### 2.6.2. Determination of Ferric Reducing Antioxidant Power (FRAP)

FRAP was determined according to the method of Oyaizu [27] with some modifications [24]. The FRAP of each freeze-dried extract, at various concentrations, was evaluated based on their abilities to reduce the Fe^3+^ complex to Fe^2+^. An increase of the Abs values indicates an increased reducing power of the samples. An aliquot of 0.4 mL of each extract sample (concentration range 3.55–28.41 µg/mL) was mixed with 0.4 mL of 200 mM of phosphate buffer (pH 6.6) plus 0.4 mL of potassium ferricyanide (1%, *w*/*v*) and the mixture was incubated for 20 min at 50 °C. After cooling down, 0.4 mL of TCA (10%, *w*/*v*) was added, and the mixture was centrifuged at 4000× *g* for 10 min. The upper layer (1 mL) was mixed with 1 mL of deionized water plus 0.2 mL of FeCl_3_ (0.1% *w*/*v*). BHT was used for comparison at the same concentration of the extracts. The results were expressed as EC_50_ value (µg/mL), which is the concentration at which the Abs was 0.5 for reducing power and were obtained by interpolation from linear regression analysis of concentration versus Abs at 700 nm against a blank. A lower EC_50_ value was indicative of a higher antioxidant activity.

#### 2.6.3. Determination of Ferrous Ion-Chelating (FIC) Activity

Since metal chelating capacity is claimed to be of the most important mechanisms which underpin antioxidant activity [28], an FIC activity assay was performed to better characterize the antioxidant activity of the samples. FIC activity was determined according to the method of Wang et al. [28] with some modifications [24]. The chelating ability of each freeze-dried extract, at various concentrations, was evaluated by measuring the inhibition of the Fe^2+^–ferrozine complex formation. An aliquot of 100 µL of each extract sample (mg/mL) was mixed with 135 µL of methanol plus 5 µL of 2 mM FeCl_2_. The reaction was initiated by the addition of 10 µL of 5 mM ferrozine. After 10 min at room temperature, the Abs was measured at 562 nm. Methanol, instead of ferrozine solution, was used as a sample blank, which is required for error correction because of unequal color of the sample solutions. Methanol, instead of a sample solution, was used as a control. Results were expressed as relative iron chelating activity compared with the unchelated (without ferrozine) Fe^2+^ reaction, and EDTA was used as the reference standard. A lower Abs was indicative of a better FIC activity. The FIC activity was calculated as follows:

FIC activity (%) = (A_0_ − (A_1_ − A_2_))/A_0_ × 100 (2)

where A_0_ was the Abs of the control, A_1_ was the Abs of the sample or standard, while A_2_ was the Abs of the blank.

### 2.7. Statistical Analysis

All determinations were performed in triplicate and the results were expressed as means ± standard deviations (SD). One-way analysis of variance test (ANOVA) was carried out to assess and indicate any significant differences between the mean values obtained from each sample. Correlations between the tea quality parameters evaluated were obtained using Pearson’s correlation coefficient (*r*). Significance was based on a confidence level of 95% (*p* < 0.05). The statistical analysis was performed using SPSS 20.0 (SPSS Inc., Chicago, IL, USA).

## 3. Results and Discussion

### 3.1. Collection and Processing the Different Azorean C. sinensis Samples and Determination of their Catechin Profile and CAF Content

#### 3.1.1. Extraction Process

Table 1 shows the collecting time (April–November), withering time (12–43 h 50 min), withering degree (43.38–64.05%), drying times (30 min–119 h), and yield (14.89–22.26%) of different “Pai-Mu-Tan” WTs (WT1–WT5), flowers from CSA, and commercial GTs from CSS. It is well documented that the catechins present in fresh leaves of *C. sinensis* remain practically unchanged in commercial GT when appropriate manufacturing processes are employed, except for a few enzymatically catalyzed changes that occur rapidly following plucking [22]. The sample yields obtained from the studied samples are as follows: 20.02–22.26%, 14.89%, and 18.60% for WTs, flowers, and average of GTs, respectively, highlighting that WT and GT samples presented higher yield values with respect to flowers sample.

**Figure 1:**
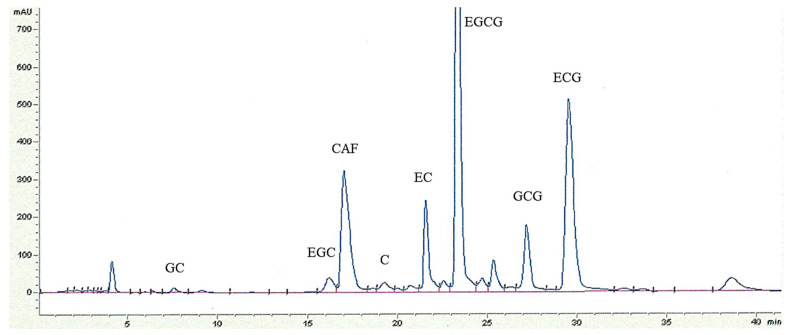
HPLC chromatogram (UV at 280 nm) of the ethyl acetate extract of commercial green tea sample of Azorean *C. sinensis* var. *sinensis* from Gorreana Tea Plantation. Peaks: GC—gallocatechin; EGC—epigallocatechin; CAF—caffeine; C—catechin; EC—epicatechin; EGCG—epigallocatechin-3-gallate; GCG—gallocatechin-3-gallate; ECG—epicatechin-3-gallate. Analytical chromatographic conditions are referred to in Methods.

The RT obtained by the HPLC analysis of the individual catechins and CAF ranged from 7.54 min for GC to 29.52 min for ECG, as illustrated in the chromatogram of GT sample (Figure 1). Table 2 shows the linear range, and regression equations, where *y* is the peak area of the analyte, *x* is the concentration in µmol/L, as well as the limits of detection and quantification of the ECDs. R is the least-squares correlation coefficient, where the range between 0.9925 and 0.9999 indicates good linearity.

The sample infusions were subjected to a sequential two-step liquid–liquid extraction, and the amounts of its individual catechins and CAF were determined by RP-HPLC/PDAD and expressed as mg/g of the sample mass on a DW basis, as reported in Table 3. This also highlighted the content of various important catechin groups.

#### 3.1.2. Catechin Profiles and CAF Content of WT, Tea Flowers, and GT Samples

The data presented in Table 3 showed that the total ECDs (EC, EGC, EGCG, and ECG) content, which has long been used as an important parameter to evaluate tea quality, was significantly high in all samples (above 111.28 mg/g of DW) but showed considerable variation, as represented by the obtained values: 111.28–197.0 (av. 161.84), 112.08, and 320.40 mg/g of DW for WTs, flowers, and average of GTs, respectively. A similar trend was observed for CAF content that showed the following values: 16.90–27.73 (av. 21.88), 6.15, and 15.98 mg/g of DW for WTs, flowers, and average of GTs, respectively, highlighting that WT and GT samples presented higher CAF values with respect to flowers sample. However, both the pre- and post-harvest stages of tea production and processing play important roles in the composition and quality of commercial teas and tea products [6]. As such, numerous studies have reported considerable variability among different tea types and brands (e.g., [1,29]). Furthermore, it should also be emphasized that comparing data obtained by several studies is quite difficult given that different raw material, extraction protocols, analytical methods, and units of measurement are used. Nevertheless, previous studies which focus on the metabolomic content of different tea varieties, leaf age, and plant tissue reported that both TCC and CAF content were, as a general rule, substantially higher in Assam cultivars than China ones, reflecting genetic variability [30,31]. They were also higher in younger shoots with respect to mature counterparts [32,33], and also relatively lower in tea flowers as compared to tea leaves [34,35,36]. On the other hand, it should be pointed out that several studies which focus on TCC variations among different cultivars reported that geographic location has a large effect on catechin levels (for a review, see [13]), which highlights the impact of the growing environments (including soil types, soil fertility, sunlight intensity, temperatures, water stress, rainfall distribution, and growth altitude) on tea’s uniqueness [6] and thus provides a possible explanation for higher values of ECDs in GT from Azorean CSS samples.

The data presented in Table 3 showed that the individual ECDs contents of the studied samples decreased as follows: EGCG > ECG ≫ EC ≫ EGC, and also showed that the content of the esterified or gallated catechins (EGCG + ECG + GCG) was significantly higher in all samples with respect to non-esterified catechins (C + EC +EGC + GC), presenting the following values: 100.83–189.93 (av. 154.06) mg/g of DW for WTs, and the values of 111.93 and 312.31 mg/g of DW for flowers and average of GTs, respectively. EGCG content was, in general, higher in the average of GTs (198.35) than in WTs (57.92–133.68, av. 103.67) followed by flowers which had a value of 68.94 mg/g of DW. EGCG plus ECG levels, which have long been considered as a key factor affecting the antioxidant activity of tea extracts [37], presented the following values: 97.0–184.52 (av. 150.26), 100.67, and 287.60 mg/g of DW for WTs, flowers, and average of GTs, respectively, that accounted for approximately 87–95% of their ECDs content, thus revealing its potential as a natural resource with high nutraceutical value. The other esterified catechin (GCG) exhibited significantly lower levels, presenting the following values: 1.25–5.84 (av. 3.76), 11.26, and 24.71 mg/g of DW for WTs, flowers, and average of GTs, respectively. Relative to the total non-esterified catechin content, the main contributor in all samples was the EC that presented the values of 8.34–12.97 (av. 10.21), 10.34, and 27.58 mg/g of DW for WTs, flowers, and average of GTs, respectively. The remaining non-esterified catechin, GC, C, and EGC, presented the values of 2.13–4.44, 1.01–2.95, and 0.96–2.23 mg/g of DW, respectively, for WTs and 0.81, 8.35, and 1.07 mg/g of DW in flowers and 0.46, 4.45, and 5.22 mg/g of DW in the averaged GTs, respectively. Zhao et al. [38] also found the order EGCG > ECG ≫ EC ≫ EGC for commercial Chinese GT samples, however, they reported significantly lower values for the TCC with respect to our results, which may be related to differences in terms of the extraction/analysis methodologies employed and by genetic, geographic location, cultivation, plucking season, processing, and storage conditions.

Our results on the catechin compositions of the studied Azorean tea samples showed that the dihydroxy (EC + ECG) to trihydroxy (EGC + EGCG) catechins ratio (CATRAT) was 0.44–0.88 for WTs and around 0.6 for both flowers and the averaged GTs. Sabhapondit et al. [31] reported similar CATRAT for eleven Assam cultivars grown in northeast India that ranged between 0.3 and 0.5. Previous studies on different tea cultivars from other regions reported that catechin compositions, such as TCC and CATRAT, could be used as markers to differentiate the China variety from Assam (for a review see [39]). Our results revealed that, in both Azorean tea varieties, the ECG was the second major catechin, possibly due to genetic variation and differences in the environment (such as pedoclimatic factors) and agricultural practices, or a combination of these factors.

Concerning the ECDs profiles defined in flowers from Azorean CSA, Lin et al. [35] also reported appreciable amounts of total ECDs content in flowers from ten different tea species, but with lower amounts with respect to our results, which may be related with differences in terms of the extraction/analysis methodologies employed as well as geographic location and different processing conditions. According to Sun et al. [40], the catechin content dynamically changed during tea flowering and the authors also described, for the first time, the function of genes related to catechins biosynthesis in tea flowers. Furthermore, according to Chen et al. [41], different tea floral organs also express different catechin profiles.

#### 3.1.3. Impact of Plucking Season and Tea Processing on Catechin Profiles and CAF Content

Regarding the seasonal and withering effects on the studied samples, the summer average GTs, under the lower withering time (12 h), presented the highest value (Table 3) of ECDs content (320.40 mg/g of DW). The WTs also showed higher ECDs content in summer (WT3 and WT4) from July, with values of 193.99 and 193.58 mg/g of DW, respectively, and in early autumn (WT5) (October is still a hot month in Azores Islands) with a value of 197.0 mg/g of DW, with respect to spring (WT1 from April and WT2 from May) with values of 113.33 and 111.28 mg/g of DW, respectively. A different pattern was found observed for CAF content that presented, in general, higher values in WTs with respect to GTs, with the highest value of 27.73 mg/g of DW (WT4) being observed in summer and lower values for the other WTs 16.90–21.68 (av. 20.42), and 6.15 and 15.98 mg/g of DW for flowers and the average of GTs, respectively. On the other hand, the CAF content increased with lower withering time in summer, 21.68 (WT3—withering time 44 h) versus 27.73 mg/g of DW (WT4—withering time 23 h) that shows a significant difference, but there was an inconsistent pattern in spring and autumn. However, in GTs (withering time 12 h), the resultant CAF value was lower, 15.98 mg/g of DW, and this may be explained by different variety and processing conditions. Other authors reported similar seasonal [13] and withering [20] effects on tea catechins and CAF content. Our findings on seasonal variations are also well corroborated by several studies reporting that the ECDs content of both China and Assam tea varieties increased during warmer months when the solar radiation (in particular UV-B radiation) and average day temperatures are the highest, as compared to the cold months [6,33]. Concerning the biochemical changes during withering, Tomlins and Mashingaidze [20] reported that ECDs decrease with increased withering times through the enhanced activity of the polyphenol oxidase enzyme, and that the increase in CAF content during withering appears to be related to amino acid metabolism.

The data presented in Table 3 also show that the same pattern found for the ECDs group of the averaged GTs and WTs (WT5 ~ WT3 ~ WT4 > WT1 ~ WT2) was also observed for the esterified catechin group, as well as for its main individual contributor (EGCG), which decreased as follows: 198.35 (GTs) > 133.68 (WT3) ~ 132.37 (WT4) ~ 131.71 (WT5) >> 62.68 (WT1) ~ 57.92 mg/of DW (WT2). Conversely, the content of total non-esterified catechins showed a different seasonal pattern in which higher values were observed in early autumn (WT5) and in spring (WT2) than in summer (WT3 and WT4). Overall, the higher values found in GTs for ECDs, esterified, and non-esterified catechins compared to the WTs can be explained by different tea variety as well as different harvesting and processing conditions.

Among the minor catechins (GCG, EGC, and C), the highest content of the esterified catechin, GCG, was observed in summer (GTs, WT3, and WT4) with values of 24.71, 4.96, and 5.84 mg/g of DW, with respect to spring (WT1 and WT2), with values of 1.25 and 3.83 mg/g of DW, respectively. This was also true with respect to autumn (WT5), which had a value of 2.93 mg/g of DW. A similar trend was observed for the non-esterified catechin C that presented in WT1 (1.01) and WT2 (1.72) in spring as compared to WT4 (2.09), WT3 (2.75), and GTs (4.45) mg/g of DW in summer.

The results also showed the potential to produce high-quality WT during autumn (WT5) in the Azores given that the WT5 sample, plucked on the October 2016 (off collecting season), withered for ca 20 h and dried for 7 h 30 min, presented the best value for total ECDs content. In fact, previous studies reported that the variation in TCC and individual catechin content among locations was far larger than those among cultivars, and that their levels show sharp seasonal fluctuations in response to temperature, day length, sun radiation, and/or UV intensity [6].

### 3.2. TPC and TFC of C. sinensis Extract Samples

Table 4 summarizes the TPC and TFC values for each sample. The TPC levels can be considered as an indirect measure of the sample’s antioxidant activities given that the basic redox mechanism of the Folin–Ciocalteu method was chosen to screen phenolics content. The TPC results, expressed in mg of GAE/g of DE, showed considerable variation and decreased in the following order: 295.37 (GTs) > 272.61 (WT5) > 269.78 (WT3) > 258.45 (WT4) > 246.03 (WT1) > 208.24 (WT2) > 125.91 (flowers), being comparable to those reported by Rohadi et al. [42], such as 308.3 and 299.9 mg of GAE/g of DE for GT and WT, respectively. Concerning the TFC levels of the samples, determined by a colorimetric method, and expressed in mg of RE/g of DE, there were also significant differences present and decreased in the following order: 69.67 (GTs) > 56.83 (WT1) > 49.50 (WT2) ~ 49.0 (WT3) ~ 48.83 (flowers) > 41.67 (WT4) > 35.17 (WT5). Other researchers, who prepared tea infusions in a similar way as in this study, also reported that GT contained more TPC and TFC than WT [17]. Zhao et al. [1] also reported that GT contains a higher level of TPC with respect to WT infusions, which could be due to the minimized degree of oxidation in young shoots, owing to inactivated enzymes during the GT steaming process. Furthermore, polyphenols in WT are gradually lost due to enzymatic and non-enzymatic oxidation reactions in the process of withering. Regarding the seasonal effects on TPC levels of the WTs, higher values were observed in summer (WT3, WT4) and the beginning of autumn (WT5) than in spring (WT1 and WT2). Conversely, contrasting results were obtained for TFC, with higher values being observed in spring (WT1) than in summer (WT4). However, spring WT2 and summer WT3 samples presented similar values, that can be explained by the long withering process (~44 h) as compared to WT1 (~26 h) and WT4 (23 h). According to Unachukwu et al. [18], the GTs presented higher values of TPC and TFC than WTs possibly due to a combination of factors such as the geographic region, genetic variability, differences in horticultural practices, harvesting, and different tea processing conditions. It is well documented that the antioxidant activity of plant extracts is usually correlated with its phenolic content, with several authors demonstrating these correlations by different statistic approaches [43].

### 3.3. In Vitro Antioxidant Activity on TPs extracts

The results of the in vitro antioxidant assays are illustrated in Table 5. The values were also compared to the synthetic antioxidants BHT and EDTA under the same assay conditions, which is a feasible approach when attempting to compare experimental results with those available in literature data.

#### 3.3.1. DPPH-FRSA Assay

The DPPH-FRSA results of the samples, expressed as EC_50_ values (µg/mL), showed considerable variation after 30 min of reaction time and decreased in the following order (lower EC_50_ indicates higher antioxidant activity): 3.6 (WT3) > 4.1 (GTs) > 4.3 (WT5) > 4.8 (WT4) > 7.7 (WT1) > 9.2 (WT2) >> 17.3 (flowers). Moreover, all samples presented better antioxidant capacity than that of BHT (EC_50_ value of 23.9 µg/mL). Significantly lower DPPH-FRSA values (higher EC_50_) were reported by Luca et al. [29] for commercial Chinese infusions of “Pai-Mu-Tan” WT (EC_50_ = 9.5–21 µg/mL) and GT (EC_50_ = 9.7–16.1 µg/mL) with respect to Azorean teas infusions. Yang et al. [44] also reported a lower value (EC_50_ value of 47.6 µg/mL) for the ethanolic extract in Chinese tea flowers with respect to the water extract of Azorean tea flowers. On the other hand, Lin et al. [35] reported that both tea flower and tea leaf extracts exhibit strong hydroxyl radical scavenging effects in the Fenton reaction system and nitric oxide-suppressing effects in lipopolysaccharide-induced RAW 264.7 cells. Furthermore, according to the literature, it should be pointed out that the antioxidant activity of tea flowers increases after budding, reaching its maximum when the petals start to split and drops to the minimum when fully bloomed [34]. On the other hand, according to Chen et al. [41], the antioxidant activity of tea flowers is primarily associated with the presence of catechins (especially EGCG and ECG) and polysaccharides.

Among the WTs, the results highlighted an inverse relationship between FRSA and withering times, as can clearly be seen in the July samples WT3 and WT4 (withered for ca 44 and 23 h, respectively). Regarding the seasonal effects on FRSA of the studied WTs, higher values were observed in summer (WT3 and WT4) and the beginning of autumn (WT5) with respect to spring (WT1 and WT2).

Knowledge of the kinetics which underpin DPPH consumption is also important because free radicals in the organism are short-lived species, which implies that the impact of a substance as an antioxidant depends on its fast reactivity towards free radicals [45]. The DPPH-FRSA results of all samples show slightly increased values with increasing reaction time (data not shown). Furthermore, they also exhibit antioxidant activity against DPPH radicals in a dose-dependent manner (data not shown).

According to the literature, catechins, as free radical scavengers, are able to stop radical chain reactions, thus preventing cellular lipids from oxidation. The relative hierarchy of effectiveness of catechins as DPPH radical scavengers is EGCG > ECG > EGC > EC > C [3,46], indicating that the presence of a gallate group at position 3 in the C ring plays the most significant role in terms of the ability to scavenge free radicals. These findings can better explain the DPPH-FRSA values of the WTs 3, 4, 5, and GTs that were observed to contain a higher content of EGCG plus ECG with respect to the other samples. However, it is well known that the antioxidant activity of tea samples not only depends on the levels of antioxidants (i.e., high TPC) but also on their chemical structure and synergistic or antagonistic effect among tea compounds.

#### 3.3.2. FRAP Assay

Information regarding FRAP activity is important because the reducing capacity of tea extracts may serve as a significant indicator of reductones, which are reported to be terminators of the free radical chain reactions present in the samples [47]. The FRAP results of the samples, expressed as an EC_50_ value (µg/mL), decreased in the following order (a lower EC_50_ indicates higher antioxidant activity): 4.8 (WT4) > 5.5 (WT5) > 6.7 (WT3) > 7.0 (GTs) > 7.6 (WT1) > 8.4 (WT2) >> 16.5 (flowers), showing a similar trend to those results obtained by DPPH–FRSA (WT 3 ~ GTs ~ WT5 ~ WT4 > WT1 > WT2 ≫ flowers), which may be explained by the fact that both assays rely on an electron/hydrogen donation mechanism. It should also be highlighted that the FRSA of WT4 and WT5 was better, or similar, to that of BHT (an EC_50_ value of 5.4 µg/mL), which is known as a strong reducing agent. Research by Xu et al. [48], focusing on the antioxidant activity of Fujian WT infusions had also reported that DPPH–FRSA and FRAP were strongly correlated.

Among the WTs, an inverse relationship between FRAP and withering times was observed, as can clearly be seen in the July samples WT3 and WT4 (withered for ca 44 and 23 h, respectively). Regarding the seasonal effects of the studied WTs on FRAP, higher values were observed in summer (WT3 and WT4) than in spring (WT1 and WT2), which can be explained by the greater sunlight intensity during summer season.

According to Zhong and Shahidi [49], the antioxidant effectiveness of EGCG in reducing ferric ion to ferrous ion, shown in vitro assays, can explain the better FRAP of the WT3, WT4, WT5, and GTs, with an EGCG content higher than the other samples.

#### 3.3.3. FIC Activity Assay

Similarly, as observed in the FRAP assay, the WT4 sample displayed the highest FIC activity (82.82%) being comparable to that of EDTA (92.22%), a potent metal-ion chelator. The FIC activity results of the other samples decreased in the following order: 65.33% (WT3) ~ 63.25% (GTs) > 58.55% (WT1) > 48.27% (WT5) ~ 47.09% (flowers) > 39.56% (WT2), a pattern similar to that of the FRSA results (WT3 ~ GTs > WT1 ≫ flowers), however, showing an inconsistent pattern for WT5. Regarding the withering and seasonal effects of the studied WTs on FIC activity, the results revealed an inverse relationship between this activity and withering times, as observed for the FRAP assay, but an inconsistent seasonal pattern.

According to the literature [3,46,49], the adjacent hydroxyl groups (*o*-diphenol) in the catechin structure can act as metal chelation sites and thus the antioxidant capacity of catechins (especially EGCG) is also attributed to their ability to chelate metal ions, such as iron and copper that catalyze free radical reactions. This can explain the better FIC activities of the WT4, WT2, WT3, and GTs that presented higher EGCG content with respect to the other samples. It should also be highlighted that the capacity of EGCG to act as a potent, nontoxic, metal chelator to pass the blood-brain barrier makes it a novel, promising, therapeutic approach for treating Alzheimer’s and Parkinson’s diseases, as well as amyotrophic lateral sclerosis, in which the accumulation of iron has been found to be correlated [3,50].

### 3.4. Pearson Correlation between Parameters

A significant correlation was found to occur among the methods used to determine the biological activities (Table 6). FRSA and TPC (*r* = 0.989) were strongly correlated. A moderate correlation was found between TPC and ECDs (*r* = 0.719), FRSA and ECDs (*r* = 0.629), FRSA and FIC activity (*r* = 0.556), and TPC and FIC activity (*r* = 0.504).

These results highlight that TPC were the major compounds that contribute to the antioxidant activities in all samples and that the main TPC are the catechins. Concerning the FIC activity, the correlations observed in the present study may suggest that other compounds, besides polyphenols, are also recognized as effective chelating agents and may also contribute to the moderate correlation.

## 4. Conclusions

The extraction protocol and analysis using a methodology based upon RP-HPLC/PDAD enabled a fast, precise, and accurate determination of the individual catechins and CAF content in different “Pai-Mu-Tan” WTs, GTs, and flowers at various plucking and processing conditions. This paper also contains key information on the antioxidant properties, TPC and TFC of the tea samples under study.

The ECDs were found to be the principal contributors to the antioxidant activities, with higher values being observed in GTs (under lower withering time) with respect to WTs. The individual ECDs decreased as follows: EGCG > ECG ≫ EC ≫ EGC, presenting higher values in summer and early autumn than in spring and an inconsistent pattern relative to the effect of withering time, revealing the strong impact of sunshine intensity compared with withering effect.

The esterified catechin (EGCG + ECG + GCG) content was significantly higher in all samples than that of non-esterified catechins (C + EC + EGC + GC). Furthermore, EGCG plus ECG levels accounting for approximately 87–95% of the total ECDs content of the samples, which are consistent with their higher antioxidant activities, and decreased as follows: GTs > WT3 ~ WT4 ~ WT5 >> WT1 ~ flowers ~ WT2, revealing a similar pattern found for the ECDs and esterified catechin groups.

The CAF content was higher in WTs with respect to GTs and flowers, and the content increased with lower withering time in summer, showing an inconsistent pattern in spring and autumn.

All the tea infusions presented strong antioxidant activities and a high content of TPC and TFC, but with lowers levels in WTs and flowers with respect to GTs, reflecting the impact of several factors, such as the genetic variability, leaf age, plant tissue, plucking time, and tea processing conditions.

Among the WTs, the WT4 sample (plucked in summer and withered for 23 h) exhibited the highest FRAP and FIC activity, as well as better FRSA and FRAP in summer and early autumn than in spring, showing an inconsistent pattern relative to the withering effect.

Our findings contribute greatly to promotion and valorization of *C. sinensis* flowers as a newly exploited, natural, material with nutraceutical and economic benefits, and/or as an alternative tea beverage due to its appreciable amounts of catechins and low CAF levels. The findings of the current study can also contribute to those databases which aim to comprehensively characterize the WT type that is becoming more common in Europe due to its delicate and sweet flavor.

## Figures and Tables

**Table 1:** Collecting time, withering time, withering degree, drying time, and yield of different “Pai-Mu-Tan” white tea (WT) and flowers (Fl) samples of Azorean *Camellia sinensis* var. *assamica* from Sete Cidades and of commercial green tea samples (GTs) of Azorean *C. sinensis* var. *sinensis* from Gorreana Tea Plantation.

Samples	Colleting Time	Withering		Drying Time ^2^	Yield (%) ^3^
		Time	Degree ^1^		
WT1	Apr, 2016	25 h 40 min	64.05	119 h	21.70
WT2	May, 2016	43 h 35 min	51.34	27 h 15 min	20.02
WT3	July, 2016	43 h 50 min	49.25	51 h 40 min	22.09
WT4	July, 2016	23 h	45.41	71 h 30min	22.26
WT5	Oct, 2016	19 h 50 min	43.38	7 h 30 min	21.29
Fl	Nov, 2016	-	-	49 h 10 min	14.89
GTs	July–Sep, 2016	12 h	46.71	30 min	18.60 *

^1^ Final withered weight/initial weight × 100. ^2^ Drying in a solar chamber, except for green tea (drying in oven at 70 °C). ^3^ Yield (%) = (dry weight/fresh weight) × 100. * Average value of five samples processed under the same conditions. WT1—dormant bud or Bhanji; WT2—first flush; WT3 and WT4—second flush; WT5—third flush.

**Table 2:** The linear range, regression equations, and the limits of detection and quantification of the major catechins derivatives (ECDs).

ECDs	Linear Range (µmol/L)	Linear Equation	*R* ^2^	LOD (µmol/L)	LOQ (µmol/L)
EGC	0.125–2.0	*y* = 115784*x* − 85881	0.9951	1.10	3.67
EC	23.4–375	*y* = 555366*x* − 23354	0.9999	1.10	3.62
EGCG	0.312–5.0	*y* = 963349*x* + 929779	0.9926	0.70	2.33
ECG	0.093–3.0	*y* = 1 × 10^6^*x* + 322547	0.9990	0.72	2.40

*R*^2^ —correlation coefficient; LOD—limit of detection (inj. vol. = 10 µL); LOQ—limit of quantification (inj. vol. = 10 µL).

**Table 3:** Catechins and caffeine content (mg/g of the sample on a dry weight basis) in different “Pai-Mu-Tan” white tea (WT) and flowers (Fl) samples of Azorean *Camellia sinensis* var. *assamica* from Sete Cidades and in commercial green tea samples (GTs) of Azorean *C. sinensis* var. *sinensis* from Gorreana Tea Plantation ^1^.

Catechins and CAF	*Camellia sinensis* Tea and Flowers Samples						
	Var. *Assamica*						Var. *Sinensis*
	WT1	WT2	WT3	WT4	WT5	Fl	GTs
GC	4.44 ± 0.38 ^a^	3.17 ± 0.39 ^b^	3.30 ± 0.23 ^b^	3.49 ± 0.26 ^b^	2.13 ± 0.21 ^c^	0.81 ± 0.10 ^d^	0.46 ± 0.09 ^d^
EGC	0.96 ± 0.09 ^c,d^	1.31 ± 0.17 ^c^	0.96 ± 0.11 ^c,d^	1.15 ± 0.10 ^c^	2.23 ± 0.11 ^b^	1.07 ± 0.09 ^c^	5.22 ± 0.22 ^a^
C	1.01 ± 0.12 ^e^	1.72 ± 0.15 ^d,e^	2.75 ± 0.24 ^c^	2.09 ± 0.12 ^c,d^	2.95 ± 0.21 ^c^	8.35 ± 0.28 ^a^	4.45 ± 0.20 ^b^
EC	9.48 ± 0.28 ^e^	12.97 ± 0.47 ^b^	8.51 ± 0.33 ^f^	8.34 ± 0.41 ^f^	11.77 ± 0.39 ^c^	10.34 ± 0.41 ^d^	27.58 ± 0.88 ^a^
EGCG	62.68 ± 2.91 ^c,d^	57.92 ± 1.02 ^d^	133.68 ± 2.83 ^b^	132.37 ± 2.71 ^b^	131.71 ± 2.37 ^b^	68.94 ± 1.87 ^c^	198.35 ± 3.01 ^a^
GCG	1.25 ± 0.20 ^g^	3.83 ± 0.25 ^e^	4.96 ± 0.21 ^d^	5.84 ± 0.31 ^c^	2.93 ± 0.28 ^f^	11.26 ± 0.29 ^b^	24.71 ± 0.53 ^a^
ECG	40.21 ± 1.83 ^c^	39.08 ± 1.21 ^c^	50.84 ± 1.31 ^b^	51.72 ± 1.47 ^b^	51.29 ± 1.51 ^b^	31.73 ± 1.19 ^d^	89.25 ± 1.93 ^a^
CAF	16.90 ± 1.18 ^c^	21.48 ± 0.44 ^b^	21.68 ± 0.38 ^b^	27.73 ± 0.61 ^a^	21.60 ± 0.21 ^b^	6.15 ± 0.12 ^d^	15.98 ± 0.25 ^c^
**CAT Groups**							
ECDs	113.33 ± 1.34 ^c^	111.28 ± 0.48 ^c^	193.99 ± 1.24 ^b^	193.58 ± 1.18 ^b^	197.00 ± 1.04 ^b^	112.08 ± 0.80 ^c^	320.40 ± 1.22 ^a^
Est. CAT	104.14 ± 1.36 ^c^	100.83 ± 0.51 ^c^	189.48 ± 1.32 ^b^	189.93 ± 1.20 ^b^	185.93 ± 1.05 ^b^	111.93 ± 0.79 ^c^	312.31 ± 1.24 ^a^
Non-est. CAT	15.89 ± 0.14 ^c^	19.17 ± 0.16 ^b^	15.52 ± 0.09 ^c^	15.07 ± 0.14 ^c^	19.08 ± 0.12 ^b^	20.57 ± 0.15 ^b^	37.71 ± 0.36 ^a^
EC + ECG	49.69 ± 0.50 ^c^	52.05 ± 0.52 ^c^	59.35 ± 0.19 ^b^	60.06 ± 0.67 ^b^	63.06 ± 0.53 ^b^	42.07 ± 0.55 ^d^	116.83 ± 0.74 ^a^
EGC + EGCG	63.64 ± 0.62 ^d^	59.23 ± 0.70 ^d^	134.64 ± 1.82 ^b^	133.52 ± 1.85 ^b^	133.94 ± 1.60 ^b^	70.01 ± 0.73 ^c^	203.57 ± 1.97 ^a^
CATRAT	0.78 ^b^	0.88 ^a^	0.44 ^e^	0.45 ^e^	0.47 ^e^	0.60 ^c^	0.57 ^c^

^1^ Values are mean ± SD (*n* = 3). The values presented for GTs are the average of five samples processed under the same conditions. Different superscript letters are significantly different (*p* < 0.05). GC—gallocatechin; EGC—epigallocatechin; C—catechin; EC—epicatechin; EGCG—epigallocatechin-3-gallate; GCG—gallocatechin-3-gallate; ECG—epicatechin-3-gallate; CAF—caffeine; CAT—catechins; ECDs (epicatechin derivatives), sum of EC, EGC, EGCG, and ECG; Est. CAT (esterified catechins), sum of EGCG, ECG, and GCG; Non-est. CAT (non-esterified catechins), sum of C, EC, EGC, and GC; CATRAT—dihydroxy-to-trihydroxy-catechin ratio = (EC + ECG) / (EGC + EGCG). WT1—dormant bud r Bhanji; WT2—first flush; WT3 and WT4—second flush; WT5—third flush.

**Table 4:** Total phenolic content (TPC) and total flavonoid content (TFC) in dry extracts (DE) of different “Pai-Mu-Tan” white tea (WT) and flowers (Fl) samples of Azorean *Camellia sinensis* var. *assamica* from Sete Cidades and of commercial green tea samples (GTs) of Azorean *C. sinensis* var. *sinensis* from Gorreana Tea Plantation ^1^.

*C. sinensis* Samples	TPC (mg GAE/g DE)	TFC (mg RE/g DE)
WT1	246.03 ± 4.60 ^d^	56.83 ± 1.04 ^b^
WT2	208.24 ± 3.36 ^e^	49.50 ± 0.87 ^c^
WT3	269.78 ± 2.39 ^b^	49.00 ± 1.80 ^c^
WT4	258.45 ± 1.46 ^c^	41.67 ± 2.08 ^d^
WT5	272.61 ± 1.07 ^b^	35.17 ± 1.61 ^e^
Fl	125.91 ± 0.83 ^f^	48.83 ± 0.58 ^c^
GTs	295.37± 5.13 ^a^	69.67 ± 1.04 ^a^

^1^ Values are mean ± SD (*n* = 3). The values presented for GTs are the average of five samples processed under the same conditions. Different superscript letters are significantly different (*p* < 0.05). GAE—gallic acid equivalentes; RE—rutin equivalents. WT1—dormant bud or Bhanji; WT2—first flush; WT3 and WT4—second flush; WT5—third flush.

**Table 5:** Free radical scavenging activity (FRSA), ferric reducing antioxidant power (FRAP), and ferrous ion-chelating (FIC) activity in dry extracts of different “Pai-Mu-Tan” white tea (WT) and flowers (Fl) samples of Azorean *Camellia sinensis* var. *assamica* from Sete Cidades and of commercial green tea samples (GTs) of Azorean *C. sinensis* var. *sinensis* from Gorreana Tea Plantation ^1^.

*C. sinensis* Samples and Control	FRSA (EC_50_^2^, µg/mL)	FRAP (EC_50_^3^, µg/mL)	FIC (%)
WT1	7.7 ± 0.23 ^c^	7.6 ± 0.14 ^d^	58.55 ± 1.46 ^b,c^
WT2	9.2 ± 0.37 ^d^	8.4 ± 0.11 ^e^	39.56 ± 2.42 ^e^
WT3	3.6 ± 0.12 ^a^	6.7 ± 0.18 ^c^	65.33 ± 0.94 ^b^
WT4	4.8 ± 0.14 ^b^	4.8 ± 0.09 ^a^	82.82 ± 1.47 ^a^
WT5	4.3 ± 0.15 ^a,b^	5.5 ± 0.15 ^b^	48.27 ± 0.70 ^c,d^
Fl	17.3 ± 0.38 ^e^	16.5 ± 0.28 ^f^	47.09 ± 1.16 ^d,e^
GTs	4.1 ± 0.13 ^a,b^	7.0 ± 0.17 ^c,d^	63.25 ± 1.78 ^b^
BHT *	23.9 ± 0.32 ^f^	5.4 ± 0.13 ^b^	-
EDTA *	-	-	92.22 ± 0.26 ^a^

^1^ Values are mean ± SD (*n* = 3). The values presented for GTs are the average of five samples processed under the same conditions. Different superscript letters are significantly different (*p* < 0.05). ^2^ Half-maximal effective concentration. ^3^ Effective concentration at which the absorbance is 0.5. * At the same concentration of the extracts. BHT—butylated hydroxytoluene; EDTA—ethylenediamine-tetraacetic disodium salt; WT1—dormant bud or Bhanji; WT2—first flush; WT3 and WT4—second flush; WT5—third flush.

**Table 6:** Correlation matrix of the studied parameters (Pearson correlations coefficients) ^1^.

Heading	ECDs	TPC	TFC	FRSA	FIC Activity
ECDs	1	-	-	-	-
TPC	**0.719**	1	-	-	-
TFC	0.411	0.163	1	-	-
FRSA	**0.629**	**0.989**	0.083	1	-
FIC activity	0.456	**0.504**	0.062	**0.556**	1

^1^ Bold values represented moderate and strong correlations. ECDs—epicatechin derivatives; TPC—total phenolic content; TFC—total flavonoid content; FRSA—free radical scavenging activity; FIC—ferrous ion-chelating.

## Data Availability

The data presented in this study are available on requested from the corresponding author.

## References

[1] Zhao C.-N., Tang G.-Y., Cao S.-Y., Xu X.-Y., Gan R.-Y., Liu Q., Mao Q.-Q., Shang A., Li H.-B. (2019). Phenolic profiles and antioxidant activities of 30 tea infusions from green, black, oolong, white, yellow and dark teas. Antioxidants.

[2] Hajiboland R. (2017). Environmental and nutritional requirements for tea cultivation. Folia Hort..

[3] Bernatoniene J., Kopustinskiene D.M. (2018). The role of catechins in cellular responses to oxidative stress. Molecules.

[4] Khan N., Mukhtar H. (2018). Tea polyphenols in promotion of human health. Nutrients.

[5] Musial C., Kuban-Jankowska A., Gorska-Ponikowska M. (2020). Beneficial properties of green tea catechins. Int. J. Mol. Sci..

[6] Tounekti T., Joubert E., Hernández I., Munné-Bosch S. (2013). Improving the polyphenol content of tea. Crit. Rev. Plant. Sci..

[7] Derouiche S. (2020). Oxidative stress associated with SARS-Cov-2 (COVID-19) increases the severity of the lung disease: A systematic review. J. Infect. Dis. Epidemiol..

[8] Zhu Y., Xie D.-Y. (2020). Docking characterization and in vitro inhibitory activity of flavan-3-ols and dimeric proanthocyanidins against the main protease activity of SARS-Cov-2. Front. Plant. Sci..

[9] Too J.C., Kinyanjui T., Wanyoko J.K., Wachira F.N. (2015). Effect of sunlight exposure and different withering durations on theanine levels in tea (*Camellia sinensis*). Food Nutr. Sci..

[10] Chen M.-L., Zhen Y., Chen Z., Cheng. S., Chen M. (2002). Tea and health—An overview. Tea Bioactivity and Therapeutic Potential.

[11] Collings E.R., Alamar M.C., Redfern S., Cools K., Terry L.A. (2019). Spatial changes in leaf biochemical profile of two tea cultivars following cold storage under two different vapour pressure deficit (VPD) conditions. Food Chem..

[12] Zhao Y., Chen P., Lin L., Harnly J.M., Yu L., Li Z. (2011). Tentative identification, quantitation, and principal component analysis of green pu-erh, green, and white teas using UPLC/DAD/MS. Food Chem..

[13] Ahmed S., Griffin T.S., Kraner D., Schaffner M.K., Sharma D., Hazel M., Leitch A.R., Orians C.M., Han W., Stepp J.R. (2019). Environmental factors variably impact tea secondary metabolites in the context of climate change. Front. Plant. Sci..

[14] Damiani E., Bacchetti T., Padella L., Tiano L., Carloni P. (2014). Antioxidant activity of different white teas: Comparison of hot and cold tea infusions. J. Food Compos. Anal..

[15] Kosińska A., Andlauer W., Preedy V. (2014). Antioxidant capacity of tea: Effect of processing and storage. Processing and Impact on Antioxidants in Beverages.

[16] Hilal Y., Engelhardt U. (2007). Characterisation of white tea—Comparison to green and black tea. J. Verbr. Lebensm..

[17] Jeszka-Skowron M., Zgoła-Grześkowiak A. (2014). Analysis of antioxidant activity, chlorogenic acid and rutin content of *Camellia sinensis* infusions using response surface methodology optimization. Food Anal. Methods.

[18] Unachukwu U.J., Ahmed S., Kavalier A., Lyles J.T., Kennelly E.J. (2010). White and green teas (*Camellia sinensis* var. sinensis): Variation in phenolic, methylxanthine, and antioxidant profiles. J. Food Sci..

[19] Zhang L., Ho C.-T., Zhou J., Santos J.S., Armstrong L., Granato D. (2019). Chemistry and biological activities of processed *Camellia sinensis* teas: A comprehensive review. Compr. Rev. Food Sci. Food Saf..

[20] Tomlins K.I., Mashingaidze A. (1997). Influence of handling on the manufacturing and quality of black teas: A review. Food Chem..

[21] Baptista J., Lima E., Paiva L., Castro A.R. (2014). Value of off-season fresh *Camellia sinensis* leaves. Antiradical activity, total phenolics content and catechin profiles. LWT—Food Sci. Technol..

[22] Baptista J.A.B., Tavares J.F.P., Carvalho R.C.B. (1999). Comparison of catechins and aromas among different green teas, using HPLC/SPME-GC. Food Res. Int..

[23] Waterhouse A.L., Wrolstad R.E. (2002). Determination of total phenolics. Current Protocols in Food Analytical Chemistry.

[24] Paiva L., Lima E., Motta M., Marcone M., Baptista J. (2020). Variability of antioxidant properties, catechins, caffeine, L-theanine and other amino acids in different plant parts of Azorean *Camellia sinensis*. Curr. Res. Food Sci..

[25] Chang C.-C., Yang M.-H., Wen H.-M., Chern J.-C. (2002). Estimation of total flavonoid content in propolis by two complementary colorimetric methods. J. Food Drug Anal..

[26] Molyneux P. (2004). The use of the stable free radical diphenylpicrylhydrazyl (DPPH) for estimating antioxidant activity. Songklanakarin J. Sci. Technol..

[27] Oyaizu M. (1986). Studies on products of browning reactions: Antioxidative activities of products of browning reaction prepared from glucosamine. Jpn. J. Nutr. Diet..

[28] Wang T., Jónsdóttir R., Ólafsdóttir G. (2009). Total phenolic compounds, radical scavenging and metal chelation of extracts from Icelandic seaweeds. Food Chem..

[29] Luca V.S., Stan A.-M., Trifan A., Miron A., Aprotosoaie A.C. (2016). Catechins profile, caffeine content and antioxidant of *Camellia sinensis* teas commercialized in Romania. Rev. Med. Chir. Soc. Med. Nat. Iasi..

[30] Cheng H., Wei K., Wang L., Preedy V.R. (2015). The impact of variety, environment and agricultural practices on catechins and caffeine in plucked tea leaves. Processing and Impact on Active Components in Food.

[31] Sabhapondit S., Karak T., Bhuyan L.P., Goswami B.C., Hazarika M. (2012). Diversity of catechin in northeast Indian tea cultivars. Sci. World J..

[32] Lin Y.S., Tsai Y.J., Tsay J.S., Lin J.K. (2003). Factors affecting the levels of tea polyphenols and caffeine in tea leaves. J. Agric. Food Chem..

[33] Sharma V., Joshi R., Gulati A. (2011). Seasonal clonal variations and effects of stresses on quality chemicals and prephenate dehydratase enzyme activity in tea (*Camellia sinensis*). Eur. Food Res. Technol..

[34] Chen Y., Zhou Y., Zeng L., Dong F., Tu Y., Yang Z. (2018). Occurrence of functional molecules in the flowers of tea (*Camellia sinensis*) plants: Evidence for a second resource. Molecules.

[35] Lin Y.-S., Wu S.-S., Lin J.-K. (2003). Determination of tea polyphenols and caffeine in tea flowers (*Camellia sinensis*) and their hydroxyl radical scavenging and nitric oxide suppressing effects. J. Agric. Food Chem..

[36] Paiva L., Lima E., Motta M., Baptista J. (2019). The surplus value of Azorean *Camellia sinensis* flowers as an important contributor affecting the nutraceutical benefits of green tea quality. Pharm. Pharmacol. Int. J..

[37] Lee L.-S., Kim S.-H., Kim Y.-B., Kim Y.-C. (2014). Quantitative analysis of major constituents in green tea with different plucking periods and their antioxidant activity. Molecules.

[38] Zhao C., Li C., Liu S., Yang L. (2014). The galloyl contributing to main antioxidant capacity of tea made from *Camellia sinensis* in China. Sci. World J..

[39] Kottawa-Arachchi J.D., Gunasekare M.T.K., Ranatunga M.A.B. (2019). Biochemical diversity of global tea [*Camellia sinensis* (L.) O. Kuntze] germplasm and its exploitation: A review. Genet. Resour. Crop. Evol..

[40] Sun L., Wang Y., Ding Z., Liu F. (2019). The dynamic changes of catechins and related genes in tea (*Camellia sinensis*) flowers. Acta Physiol. Plant..

[41] Chen D., Chen G., Sun Y., Zeng X., Ye H. (2020). Physiological genetics, chemical composition, health benefits and toxicology of tea (*Camellia sinensis* L.) flowers: A review. Food Res. Int..

[42] Rohadi R., Lelita D.I., Putri A.S. (2019). Antioxidant capacity of white tea (*Camelia sinensis*) extract: Compared to green, oolong and black tea. IOP Conf. Ser. Earth Environ. Sci..

[43] Ulewicz-Magulska B., Wesolowski M. (2019). Total phenolic contents and antioxidant potential of herbs used for medical and culinary purposes. Plant. Foods Hum. Nutr..

[44] Yang Z., Tu Y., Baldermann S., Dong F., Xu Y., Watanabe N. (2009). Isolation and identification of compounds from the ethanolic extract of flowers of the tea (*Camellia sinensis*) plant and their contribution to the antioxidant capacity. Food Sci. Technol..

[45] Lobo V., Patil A., Phatak A., Chandra N. (2010). Free radicals, antioxidants and functional foods: Impact on human health. Pharmacogn. Rev..

[46] Rice-Evans C.A., Miller N.J., Paganga G. (1997). Antioxidant properties of phenolic compounds. Trends Plant. Sci..

[47] Duh P.-D. (1998). Antioxidant activity of burdock (*Arctium lappa* Linné): It’s scavenging effect on free radical and active oxygen. J. Am. Oil Chem. Soc..

[48] Xu P., Chen L., Wang Y. (2019). Effect of storage time on antioxidant activity and inhibition on α-amylase and α-glucosidase of white tea. Food Sci. Nutr..

[49] Zhong Y., Shahidi F. (2011). Lipophilized epigallocatechin gallate (EGCG) derivatives as novel antioxidants. J. Agric. Food Chem..

[50] Mandel S., Amit T., Reznichenko L., Weinreb O., Youdim M.B.H. (2006). Green tea catechins as brain-permeable, natural iron chelators-antioxidants for the treatment of neurodegenerative disorders. Mol. Nutr. Food Res..

